# What do Danish children eat, and does the diet meet the recommendations? Baseline data from the OPUS School Meal Study

**DOI:** 10.1017/jns.2015.17

**Published:** 2015-08-26

**Authors:** Rikke Andersen, Anja Biltoft-Jensen, Tue Christensen, Elisabeth W. Andersen, Majken Ege, Anne V. Thorsen, Vibeke K. Knudsen, Camilla T. Damsgaard, Louise B. Sørensen, Rikke A. Petersen, Kim F. Michaelsen, Inge Tetens

**Affiliations:** 1 Division of Nutrition, National Food Institute, Technical University of Denmark, Søborg, Denmark; 2 Department of Applied Mathematics and Computer Science, Technical University of Denmark, Kgs. Lyngby, Denmark; 3 Department of Nutrition, Exercise and Sports, Faculty of Science, University of Copenhagen, Copenhagen, Denmark

**Keywords:** Whole diet, Food-based dietary guidelines, Nutrition recommendations, School lunch, AR, acceptable reporter, DANSDA, Danish National Survey of Diet and Physical Activity, EI, energy intake, E%, percentage energy, FBDG, Food-Based Dietary Guidelines 2013, NNR2012, Nordic Nutrition Recommendations, OPUS, Optimal well-being, development and health for Danish children through a healthy New Nordic Diet, OR, over-reporter, UR, under-reporter, WebDASC, Web-based Dietary Assessment Software for Children

## Abstract

A child's diet is an important determinant for later health, growth and development. In Denmark, most children in primary school bring their own packed lunch from home and attend an after-school care institution. The aim of the present study was to evaluate the food, energy and nutrient intake of Danish school children in relation to dietary guidelines and nutrient recommendations, and to assess the food intake during and outside school hours. In total, 834 children from nine public schools located in the eastern part of Denmark were included in this cross-sectional study and 798 children (95·7 %) completed the dietary assessment sufficiently (August–November 2011). The whole diet was recorded during seven consecutive days using the Web-based Dietary Assessment Software for Children (WebDASC). Compared with the food-based dietary guidelines and nutrient recommendations, 85 % of the children consumed excess amounts of red meat, 89 % consumed too much saturated fat, and 56 % consumed too much added sugar. Additionally 35 or 91 % of the children (depending on age group) consumed insufficient amounts of fruits and vegetables, 85 % consumed insufficient amounts of fish, 86 % consumed insufficient amounts of dietary fibre, 60 or 84 % had an insufficient Fe intake (depending on age group), and 96 % had an insufficient vitamin D intake. The study also showed that there is a higher intake of fruits and bread during school hours than outside school hours; this is not the case with, for example, fish and vegetables, and future studies should investigate strategies to increase fish and vegetable intake during school hours.

Danish children and adolescents have similar lifestyle-related health issues as other children in many Western countries have, with an increasing prevalence of being overweight^(^^1^^)^. Weight issues and obesity among Danish school children are major concerns in which social inequalities have been documented to play an important role^(^^2^^)^. It has been proposed that a diet not fulfilling the official dietary guidelines^(^^3^^)^ may contribute to future generations with a higher prevalence of lifestyle-related diseases^(^^4^^,^^5^^)^.

In Denmark, most children in primary school bring their own packed lunch from home^(^^6^^–^^8^^)^. This packed lunch typically consists of open sandwiches based on rye bread with meat products supplemented with fruit and/or vegetables. Typically, the children drink milk or water with their meal^(^^6^^)^. A previous survey showed that food stands or canteens are present at 64 % of Danish primary and secondary schools and the most frequent food items available from these stands are sandwiches, toast or sausage with bread, yoghurt, fruit and cheese sticks^(^^9^^)^. Only 17 % of schools have a policy on nutrition^(^^9^^)^. Moreover, there is no compulsory regulation at the national level concerning the provision and quality of public school meals in Denmark^(^^10^^)^. Vending machines, however, are rarely present at Danish schools (0·2 %) and are not present at all at after-school care institutions^(^^9^^)^. Primary school children often have snacks brought from home before lunch and after school hours; most primary school children attend an after-school care institution, where an afternoon snack is served. The quality of this afternoon snack varies^(^^11^^)^.

The school and after-school care institutions are important settings for improving the diet of children since they eat lunch and two snack meals at school 5 d per week. Earlier studies have shown that Danish children aged 8–10 years consume 40–45 % of their daily energy intake (EI) during school hours and after school activities^(^^12^^)^. Direct comparisons of lunch types among children in other Western countries are not straightforward because the traditional lunch eaten in Denmark based on open rye sandwiches differs from what is normally eaten in other countries. Due to the importance of children's health, there is a large focus on the dietary quality of children's lunch^(^^13^^–^^18^^)^.

Denmark has two sets of official dietary recommendations: the Danish Food-Based Dietary Guidelines 2013 (FBDG)^(^^3^^)^ and the Nordic Nutrition Recommendations (NNR2012)^(^^19^^)^. The FBDG communicate the concept of a healthy diet to the public and include ten specific key messages to healthy people above 2 years of age and are usually expressed as amounts of foods per d per 10 MJ^(^^5^^,^^20^^)^. The NNR2012 provide a basis for evaluating the intake of nutrients in groups of healthy individuals^(^^19^^,^^20^^)^.

The aim of the present study was to evaluate the food, energy and nutrient intake of Danish school children in relation to the dietary guidelines and nutrient recommendations, and to assess the food intake during and outside school hours.

## Subjects and methods

### Design

The OPUS (Optimal well-being, development and health for Danish children through a healthy New Nordic Diet) centre carried out a School Meal Study, which was a cluster-randomised controlled unblinded cross-over study and the present study was a cross-sectional study based on baseline data from the OPUS School Meal Study. The overall design of the OPUS School Meal Study has been described in detail elsewhere^(^^21^^)^. Written informed consent was obtained from parents or custody holders (from here on referred to as ‘parent’) of the children. The study protocol was approved by the Danish National Committee on Biomedical Research Ethics (H-1-2010-124) and the trial was registered in the database www.clinicaltrials.gov (no. NCT 01457794)^(^^21^^)^.

### Subjects

Initially contact was established with thirty-nine schools. Inclusion criteria for each school were location in the eastern part of Denmark (Zealand and Lolland-Falster), at least four classes in total at 3rd and 4th grade, suitable kitchen facilities available for food production during the school day, and a high motivation for participation as determined by the study team. Out of the thirty-nine schools contacted, nineteen schools did not wish to participate, six schools did not fulfil the inclusion criteria, three schools expressed interest too late for participation, two schools were excluded for logistical reasons, and nine schools (forty-six school classes, 3rd and 4th grade) were included in the OPUS School Meal Study. A total of 1021 children were invited, and 834 children were included in this cross-sectional study. Exclusion criteria for the children were diseases or conditions that might obstruct the measurements or put the children at risk if eating the OPUS school meals served in the intervention period. The recruitment procedure, inclusion and exclusion criteria are described in detail elsewhere^(^^21^^)^.

### Background information

Together with at least one parent, each child underwent a 2-h in-depth interview on various background variables by a trained interviewer (including instructions in using the dietary assessment tool), either at the school or in the home^(^^21^^)^. The educational level of the household was categorised according to the standard classifications of Statistics Denmark, i.e. as the highest level of education achieved by a parent in the household. The variable was divided into six groups depending on the educational level (lower secondary education, upper secondary education or equivalent, vocational education, short higher education, bachelor's degree or equivalent, master's degree or higher education). This cross-sectional baseline study is part of a large School Meal Study (intervention), which has been described elsewhere^(^^21^^)^. Many other sociodemographic descriptors were collected; however, only the educational level of the household is described here, as it was the variable included in the statistical analyses of the present paper.

### Dietary assessment

The whole diet was recorded during seven consecutive days using the interactive Web-based Dietary Assessment Software for Children (WebDASC) developed for the purpose and validated during the OPUS School Meal Pilot Study^(^^22^^–^^24^^)^. WebDASC is a self-administered Internet-based interactive food record tool to be used by 8- to 11-year-old children with or without the support of a parent^(^^22^^)^. WebDASC is an intuitive, cost-effective and engaging dietary assessment method with special consideration given to age-appropriate design issues, where an animated armadillo figure guides the respondents through six daily eating occasions^(^^22^^)^. WebDASC was well accepted by the children and their parents^(^^22^^)^. WebDASC was validated by measuring EI against energy expenditure^(^^24^^)^ and by comparing fruit, juice and vegetable intake to plasma carotenoid concentrations^(^^23^^)^. Both the parents and the children were instructed in how to use the dietary assessment tool by actually trying the tool together with the trained interviewer, who also took them through how to log-in and through all steps of the dietary assessment. Reminder emails were sent to the parents if the dietary assessments were not performed on a daily basis during the 7-d assessment periods. Reminder telephone calls were performed when the dietary assessment was not initiated during the first days of the assessment period, or if the registration of the diet stopped during the registration week. A telephone hotline was also available during the period for the families in case of questions. The dietary assessment was performed during the weeks prior to the clinical measurements as described elsewhere^(^^21^^)^. As the schools joined the study one by one, children from the first school performed their dietary assessments in August 2011 and children from the last school performed their dietary assessments in November 2011. Due to ethical reasons and in order to avoid exclusions based on social differences, children without a computer or Internet were also able to participate in the study. Six children without access to a computer or the Internet filled in a paper version of a 7-d pre-coded food record, which was based on the food record used in the Danish National Survey of Diet and Physical Activity (DANSDA) 2003–2008^(^^25^^)^. The data were transferred to WebDASC by a dietitian.

### Estimation of dietary intake

The intake data were processed by the in-house-developed General Intake Estimation System (GIES), a system originally developed for the DANSDA^(^^25^^)^, which interprets the recorded consumption into ingredients that are the basis for the further calculations and estimations of intake of food, energy and nutrients for each individual. For these calculations, intake was directly collected by querying the WebDASC data tables. GIES used recipes developed for WebDASC and the nutrient data originating from the Danish Food Composition Databank, revision 7^(^^26^^)^. Dietary intake was estimated for each child as an average of the recorded days. The dietary intake was estimated and reported in thirteen food groups (milk and milk products, cheese and cheese products, bread and other cereal products, potatoes and potato products, vegetable and vegetable products, fruit and fruit products, meat and meat products, poultry and poultry products, fish and fish products, eggs, fats, sugar and candy (i.e. various types of candy, chocolate, marzipan, honey), beverages (excluding milk)), EI, the energy distribution (fat, total carbohydrate, added sugar, protein), nine macronutrients (total fat, saturated fat, *trans*-fatty acids, monounsaturated fat, polyunsaturated fat, protein, carbohydrate, added sugar (sugar and sugar from candy and chocolate), dietary fibre), and nineteen micronutrients (vitamins A, D, E, B_1_, B_2_, niacin, B_6_, folate, B_12_ and C; and minerals Ca, P, Mg, Fe, Zn, I, Se, Na and K). Intake from supplements was not included in the intake calculations.

Food was defined as solid food and liquids consumed as food (for example soups and yoghurt). Energy density was calculated as energy (kJ) divided by weight (g) for food and beverages separately. Beverages were defined as both energy-containing (for example milk, sweetened drinks and juice) and non-energy-containing (for example water and artificially sweetened drinks).

### Under-, acceptable and over-reporters

Under-reporters (UR), acceptable reporters (AR) and over-reporters (OR) were determined by the subjects’ EI:BMR ratio (where EI = mean reported EI) and classified by cut-offs suggested by Black^(^^27^^)^: UR: EI:BMR ≤ 1·05; AR: EI:BMR = 1·06–2·27; and OR: EI:BMR ≥ 2·28 using a physical activity level of 1·55. BMR was calculated from descriptive equations (Oxford prediction) using height, weight, sex and age^(^^28^^)^.

### Statistical analysis

Analyses included standard descriptive statistics. All means and medians include both eaters and non-eaters (with zero-intake).

Hierarchical mixed models were performed to investigate the effect of sex and grade (3rd or 4th grade) on the intake for all food groups, EI, energy distribution, energy density, and micro- and macronutrients. As the children were nested in classes (the whole class was randomised together), and the classes were nested in schools, the models included two random effects (class and school). The models also included fixed effects: sex (boy/girl); grade (3rd/4th); season (autumn/winter/spring); BMI (in four groups of approximately similar size, BMI ≤ 15·6, 15·6 < BMI ≤ 16·6, 16·6 < BMI ≤ 18·6, BMI > 18·6 kg/m^2^); and household education (lower secondary education, upper secondary education or equivalent, vocational education, short higher education, bachelor's degree or equivalent, master's degree or higher education).

Some children had zero intake for some food groups (cheese, potatoes, poultry or fish), and these semi-continuous outcomes were analysed using bootstrap methods^(^^29^^)^, and cluster bootstrap sampling of the schools (the highest level of data)^(^^30^^)^ was performed with 10 000 replications. The estimated means for sex and grade were calculated and the confidence limits were the 2·5 and 97·5 % percentiles in the bootstrap samples.

UR and OR were included in all models together with the AR; this effect of reporting (UR, AR, OR) was included in separate hierarchical mixed models.

The assumptions underlying the models were tested using residual plots and quantile–quantile (QQ) plots.

The outcomes are all continuous variables, and some variables (fruit, meat, eggs, fats, sugar and candy, beverages, all micronutrients) were transformed using the logarithm (log_2_). All transformed variables were back-transformed using the anti-log when presenting the results.

The dietary intake for each child was divided into during school hours and outside school hours and the differences were assessed. As in previous analyses, the trial was cluster-randomised, resulting in two random effects: a class effect and a school effect. The assumptions underlying the models were tested using residual plots and QQ plots and when the assumptions were not fulfilled the outcomes were analysed using bootstrap methods as described above.

SAS version 9.3 and Stata version 12 (StataCorp) were used for all statistical analyses. The significance level chosen was *P* < 0·05.

## Results

In total, 834 children were included in this cross-sectional study. Of the children, ten withdrew before the dietary assessment began (reasons for dropouts are described elsewhere^(^^21^^)^, twenty-four fulfilled the dietary assessment insufficiently (0–3 d) and were excluded, and two were excluded due to recording extreme intake (EI below 2000 or above 19 000 kJ/d); thus 798 children (95·7 %) fulfilled the dietary assessment sufficiently (4–7 d) – 381 (47·7 %) girls and 417 (52·3 %) boys. The characteristics of the children are shown in Table 1.

**Table 1.** Characteristics of the children (Mean values and standard deviations, or number of participants and percentages)

			Boys				Girls			
	All		3rd grade		4th grade		3rd grade		4th grade	
	Mean	sd	Mean	sd	Mean	sd	Mean	sd	Mean	sd
Participants										
*n*	798		201		216		185		196	
%	100		48		52		49		51	
Age (years)	9·98	0·6	9·5	0·4	10·5	0·4	9·4	0·4	10·4	0·4
Weight (kg)*	35·0	7·1	32·7	6·2	37·9	7·3	32·5	5·9	36·7	7·2
Height (cm)†	142·5	7·1	139·6	6·3	145·7	6·7	138·8	6·3	145·2	6·3
BMI (kg/m^2^)*	17·1	2·4	16·7	2·3	17·7	2·5	16·8	2·3	17·3	2·5
Under-reporters										
*n*	70		14		18		16		22	
%	9		7		9		9		11	
Acceptable reporters										
*n*	708		181		192		166		169	
%	89		91		89		90		88	
Over-reporters										
*n*	14		5		5		2		2	
%	2		3		2		1		1	

* *n* 792.

† *n* 793.

### The children's diet in relation to the Danish Food-based Dietary Guidelines and the Nordic Nutrition Recommendations

The observed unadjusted mean or median intakes of food groups, energy, macronutrients and micronutrients are shown in Tables 2–4. In Table 5 the proportions of the children that meet the Danish FBDG^(^^3^^)^ or the NNR2012^(^^19^^)^ are shown.

**Table 2.** Daily food intake in Danish children aged 8–11 years (*n* 798) and the effect of sex and grade on the intake* (Observed unadjusted medians and 10th and 90th percentiles)

	All			Girls			Boys				*P*	
Food group (g/d)	Median	10th percentile	90th percentile	Median	10th percentile	90th percentile	Median	10th percentile	90th percentile	*n*_0_	Sex	Grade
Milk, milk products	340	132	626	325	125	590	355	138	676	0	0·008	0·06
Cheese, cheese products	16	5·0	39	15	5·1	38	17	4·9	39	15	0·27	0·99
Bread, other cereal products	202	141	284	186	134	255	224	151	301	0	<0·0001	0·15
Potatoes, potato products	36	0·8	94	34	1·0	90	37	0·7	105	49	0·02	0·41
Vegetables, vegetable products	126	54	227	126	59	222	126	51	230	0	0·96	0·43
Fruit, fruit products	126	38	244	131	45	243	124	29	244	1	0·02	0·19
Meat, meat products	87	42	160	76	38	140	97	47	172	0	<0·0001	0·77
Poultry, poultry products	17	0	56	16	0	52	18	0	61	125	0·02	0·47
Total fish, fish products	12	0	45	12	0	40	12	0	50	195	0·06	0·72
Fat fish, fat fish products	1·5	0	27	2·1	0	27	0	0	29	387	0·20	0·03
Lean fish, lean fish products	2·5	0	28	2·5	0	24	2·2	0	30	342	0·08	0·50
Eggs	14	3·9	33	13	4·2	30	14	3·9	36	7	0·54	0·63
Fats	23	12	40	21	11	36	26	12	43	0	<0·0001	0·17
Sugar and candy	41	15	74	38	15	69	43	17	78	1	0·003	0·60
Beverages (excluding milk)	707	397	1176	664	393	1056	765	413	1263	0	<0·0001	0·16

*n*_0_, Number of children with zero intake (medians include children with zero intakes).

* Analysed by hierarchical mixed models, controlled for random effects (child, class, school) and fixed effects (sex, grade, season, BMI, household education).

**Table 3.** Daily energy intake, dietary content and macronutrient intake in Danish children aged 8–11 years (*n* 798) and the effect of sex and grade on the intake* (Observed unadjusted mean values and standard deviations)

	All		Girls		Boys		*P*	
Energy, macronutrient	Mean	sd	Mean	sd	Mean	sd	Sex	Grade
Energy (MJ/d)	7·6	1·7	7·0	1·4	8·1	1·8	<0·0001	0·20
Total fat (E%)	32	4·3	32	4·2	32	4·4	0·46	0·71
Saturated fat (E%)	13	2	13	2·1	13	2·2	0·67	0·92
*Trans*-fatty acids (E%)	0·7	0·2	0·7	0·2	0·7	0·2	0·79	0·44
Monounsaturated fat (E%)	11	2	11	2·0	11	2·0	0·97	0·62
Polyunsaturated fat (E%)	5	0·9	5	0·9	5	0·9	0·85	0·36
Total carbohydrate (E%)	53	4·8	53	4·7	53	4·9	0·48	0·61
Added sugar (E%)	11	4·5	11	4·2	11	4·7	0·67	0·17
Protein (E%)	15	2·2	15	2·3	16	2·1	0·79	0·81
Total fat (g/d)	66	18	60	16	70	19	<0·0001	0·32
Saturated fat (g/d)	26	8	24	7	28	8	<0·0001	0·30
*Trans*-fatty acids (g/d)	1·4	0·5	1·3	0·5	1·5	0·6	<0·0001	0·16
Monounsaturated fat (g/d)	24	7	22	6	25	7	<0·0001	0·41
Polyunsaturated fat (g/d)	10	3	9	3	10	3	<0·0001	0·07
Total carbohydrate (g/d)	244	58	226	49	260	60	<0·0001	0·19
Added sugar (g/d)	50	26	47	21	53	29	0·0002	0·65
Protein (g/d)	68	17	63	15	73	17	<0·0001	0·30
Dietary fibre (g/d)	18	5·5	17	4·7	19	5·9	<0·0001	0·002

E%, percentage energy.

* Analysed by hierarchical mixed models, controlled for random effects (child, class, school) and fixed effects (sex, grade, season, BMI, household education).

**Table 4.** Daily micronutrient intake in Danish children aged 8–11 years (*n* 798) and the effect of sex and grade on the intake* (Observed unadjusted mean values and standard deviations)

	All		Girls		Boys		*P*	
Micronutrient	Mean	sd	Mean	sd	Mean	sd	Sex	Grade
Vitamin A (RE/d)	837	531	766	470	901	573	0·008	0·03
Vitamin D (μg/d)	2·7	2·7	2·5	2·4	2·9	3·0	0·02	0·29
Vitamin E (α-TE/d)	5·9	2·0	5·6	1·8	6·1	2·1	0·002	0·50
Vitamin B_1_ (mg/d)	1·1	0·3	1·0	0·3	1·2	0·3	<0·0001	0·07
Vitamin B_2_ (mg/d)	1·4	0·5	1·3	0·4	1·5	0·5	<0·0001	0·02
Niacin (NE/d)	23	5·9	21	5·5	24	6·0	<0·0001	0·25
Vitamin B_6_ (mg/d)	1·2	0·3	1·1	0·3	1·3	0·3	<0·0001	0·15
Folate (μg/d)	241	84	229	89	253	77	<0·0001	0·16
Vitamin B_12_ (μg/d)	4·7	2·3	4·3	2·1	5·1	2·4	<0·0001	0·03
Vitamin C (mg/d)	77	39	78	38	76	39	0·18	0·33
Ca (mg/d)	909	296	852	268	961	310	<0·0001	0·11
P (mg/d)	1260	324	1169	284	1343	336	<0·0001	0·02
Mg (mg/d)	262	69	243	59	280	72	<0·0001	0·008
Fe (mg/d)	8·6	2·2	7·9	1·9	9·3	2·3	<0·0001	0·07
Zn (mg/d)	9·6	2·6	8·8	2·3	10	2·6	<0·0001	0·11
I (μg/d)	227	104	207	95	246	109	<0·0001	0·02
Se (μg/d)	39	12	36	11	41	12	<0·0001	0·16
Na (g/d)	3·0	0·8	2·7	0·7	3·2	0·8	<0·0001	0·17
K (g/d)	2·5	0·6	2·3	0·6	2·6	0·6	<0·0001	0·01

RE, retinol equivalents; TE, tocopherol equivalents; NE, niacin equivalents.

* Analysed by hierarchical mixed models, controlled for random effects (child, class, school) and fixed effects (sex, grade, season, BMI, household education).

**Table 5.** Percentage of children in relation to the Danish Food Based Dietary Guidelines (FBDG) or Nordic Nutrition Recommendations (NNR2012)

Intake*	*n*	%	FBDG^(^^3^^)^ or NNR2012^(^^19^^)^
Milk and milk products			250–500 ml/d†
<250 ml/d	126	16	
250–500 ml/d	329	41	
>500 ml/d	343	43	
Cheese and cheese products			Maximum 25 g/d†
≤25 g/d	469	59	
>25 g/d	329	41	
Fruit and vegetables			4–9 years: 300–500 g fruit and vegetables/d‡
≤9 years: <300 g/d	138	35	≥10 years: 600 g fruit and vegetables/d‡
≤9 years: 300–500 g/d	187	47	
≤9 years: >500 g/d	71	18	
≥10 years: <600 g/d	365	91	
≥10 years: ≥600 g/d	37	9	
Meat and meat products (red)			Maximum 500 g/week
≤500 g/week	121	15	
>500 g/week	677	85	
Total fish and fish products			Minimum 350 g fish/week, 200 g fat fish/week
≥350 g/week	120	15	
<350 g/week	678	85	
Fat fish, and fish products			
≥200 g/week	118	15	
<200 g/week	680	85	
Total fat			25–40 E%
<25 E%	45	6	
25–40 E%	728	91	
>40 E%	25	3	
Saturated fat			<10 E%
<10 E%	92	11	
≥10 E%	706	89	
Protein			10–20 E%
<10 E%	2	0·3	
10–20 E%	771	97	
>20 E%	25	3	
Carbohydrates			45–60 E%
<45 E%	35	4	
45–60 E%	716	90	
>60 E%	47	6	
Added sugars			<10 E%
<10 E%	353	44	
≥10 E%	445	56	
Dietary fibre (g/d)			≥3 g/MJ
<3 g/MJ	686	86	
≥3 g/MJ	112	14	
Vitamin D (μg/d)			10 µg/d
≤10 µg/d	765	96	
>10 µg/d	33	4	
Fe (mg/d)			6–9 years: 9 mg/d
≤9 years: <9 mg/d	238	60	10–13 years 11 mg/d
≤9 years: ≥9 mg/d	158	40	
≥10 years: <11 mg/d	338	84	
≥10 years: ≥11 mg/d	64	16	

E%, percentage energy.

* The intake is per 10 MJ (as the recommendations are), except for dietary fibre (per MJ), vitamin D (μg/d) and Fe (mg/d).

† Milk and cheese are not quantified in the FBDG; Beck *et al.*^(^^31^^)^ is used instead.

‡ About half fruit and half vegetables is recommended.

#### Food groups

The intake of fruit and vegetables was below the recommended 300 g/d for 35 % of the children ≤9 years, and below the recommended 600 g/d for 91 % of the children ≥10 years (Table 5). It is recommended to eat approximately half vegetables and half fruit^(^^5^^)^, which both girls and boys did; however, the intake was too low (Table 2).

It is recommended to eat at least 350 g of fish per week, and of that, 200 g should be fatty fish; 85 % of the children did not eat enough fish (Table 5). The median intake of fish and fish products was 12 (10th, 90th percentiles 0, 45) g/d (Table 2).

Of the children, 16 % drank less milk than recommended (<250 ml/d)^(^^31^^)^, and 41 % ate too much cheese (>25 g/d)^(^^31^^)^ (Table 5). The median intake of milk was 340 (10th, 90th percentiles 132, 626) ml/d and the intake of cheese was 16 (10th, 90th percentiles 5, 39) g/d (Table 2).

The median intake of meat and meat products was 87 (10th, 90th percentiles 42, 160) g/d. Of the children, 85 % ate more than 500 g of meat per week (red meat, poultry not included).

The most important differences between the sexes (*P* values are shown in Table 2) are seen for bread and other cereal products (14 %; 95 % CI −18, −11), meat (19 %; 95 % CI −25, −13), fats (15 %; 95 % CI −21, −9) and sugar and candy (13 %; 95 % −21, −5), where girls had a significantly lower intake than boys. In contrast, girls had a significantly higher intake of fruit than boys (15 %; 95 % CI 2, 28). There was no difference between girls and boys for vegetables, eggs, cheese and fish. The only significant difference between 3rd and 4th graders (*P* values are shown in Table 2) was found for intake of fatty fish (mean difference 2 g/d; 95 % CI 0·3, 4), where 3rd graders had a higher intake than 4th graders. The differences between sex and grade were not affected by whether the children were UR, AR or OR (data not shown).

#### Energy and macronutrients

The diet of almost all children (91 %) contained between 25–40 % of energy (E%) from fat; however, the diet contained too much saturated fat (≥10 E%) for 89 % of the children (Table 5). The mean of saturated fat intake was 26 (sd 8) g/d (Table 3). The diet contained between 10 and 20 E% (recommended) from protein for 97 % of the children, and 45–60 E% (recommended) from carbohydrates for 90 % of the children, but the diet of more than half of the children (56 %) contained too much added sugar (≥10 E%) and not enough dietary fibre (<3/MJ) for 86 % of the children (Table 5). The mean EI from fat was 32 (sd 4·3) E%; 53 (sd 4·8) E% from total carbohydrate and 15 (sd 2·2) E% from protein (Table 3).

The mean EI was slightly lower than recommended by the NNR2012 for both girls and boys. The mean EI was 7·0 (sd 14) and 8·1 (sd 1·8) MJ/d for girls and boys, respectively (Table 3). Based on average weight and moderate physical activity, the NNR estimates the energy requirements for 8- to 11-year-old girls and boys to be 7·4–8·2 MJ/d and 8·2–9·4 MJ/d, respectively.

Girls had a significantly lower total EI (1·0 MJ/d; 95 % CI −1·3, −0·8); however, there were no differences between boys and girls regarding the energy distribution from total fat, total carbohydrate, added sugar and protein (*P* values are shown in Table 3). Girls had a significantly lower intake of total fat (9 g/d; 95 % CI −12, -7), saturated fat (3·7 g/d; 95 % CI −4·8, −2·6), monounsaturated fat (3·2 g/d; 95 % CI –4·2, −2·3), polyunsaturated fat (1·3 g/d; 95 % CI −1·7, −0·9) and *trans-*fatty acids (0·2 g/d; 95 % CI −0·3, −0·1), carbohydrate (32 g/d; 95 % CI −39, −24), dietary fibre (2·0 g/d; 95 % CI −2·7, 1·3) and protein (10 g/d; 95 % CI −12, −7) as well as added sugar (7 g/d; 95 % CI −10, −3) (*P* values are shown in Table 3). The only significant difference between 3rd and 4th graders was found for dietary fibre (1·3 g/d; 95 % CI 0·5, 2·1), where 3rd graders had a higher intake than 4th graders (*P* values are shown in Table 3). The differences between sex and grade were not affected by whether the children were UR, AR or OR (data not shown).

#### Micronutrients

In order to fulfil the NNR, the most critical micronutrients for Danish children are vitamin D and Fe. The intake of vitamin D was below 10 µg/d for 96 % of the children (Table 5). The intake of Fe was below 9 mg/d for 60 % of the children ≤9 years, and below 11 mg/d for 84 % of the children ≥10 years (Table 5).

Girls had a significantly lower intake than boys of all vitamins and minerals (7–15 %), except for vitamin C (*P* > 0·05) (Table 4). The 3rd graders had a higher intake than the 4th graders of vitamin A, vitamin B_2_, vitamin B_12_, P, Mg, I and K (5–12 %) (Table 4). The differences between sexes and grade were not affected by whether the children were UR, AR or OR, except for K and vitamin A, where the difference between 3rd and 4th graders changed from significant to non-significant (data not shown).

### The children's diet during and outside school hours

The intake of energy and the food groups outside of school hours (total of breakfast, dinner, and evening snack) and during school hours (total of morning snack, lunch and afternoon snack) were analysed testing the null hypothesis that the intake distributions outside and during school hours are the same. There is a significant difference between intake during and outside school hours apart from total and lean fish and fish products as well as fats.

About 44 % of the EI was consumed during school hours. The mean intake of energy was significantly (*P* < 0·0001) lower during school hours compared with the rest of the day (on average 1 MJ/d; 95 % CI 0·8, 1·2 MJ/d).

The mean intake of fruit (*P* < 0·0001) and bread (*P* < 0·0001) was significantly higher during school hours compared with the rest of the day. The median intake of fruit was 34 (10th, 90th percentiles 1·8, 106) g/d outside school hours and 93 (10th, 90th percentiles 17, 204) g/d during school hours. The median intake of bread was 96 (10th, 90th percentiles 56, 156) g/d outside school hours and 106 (10th, 90th percentiles 63, 168) g/d during school hours. No difference was found between the intake outside and during school hours for fish (total and lean) and fats; for the other food groups (milk, cheese, meat, poultry, potatoes, vegetables, eggs, sugar and candy) the intake was higher outside school hours than during school hours. The median intake of sugar and candy was 18 (10th, 90th percentiles 3·4, 45) g/d outside school hours and 13 (10th, 90th percentiles 2·3, 33) g/d during school hours.

## Discussion

In this cross-sectional study, children aged 8–11 years in the 3rd and 4th grades in general consumed too much red meat, saturated fat and added sugar, but not enough fruit and vegetables, fish, dietary fibre, Fe and vitamin D according to the FBDG and NNR2012. However, the energy distribution from total fat, total carbohydrate and protein was within the range recommended in the NNR2012^(^^19^^)^ and was similar to the energy distribution reported for the much smaller group of children of similar age (*n* 260) participating in the DANSDA 2003–2008^(^^6^^)^. About 44 % of the daily EI was consumed during the school hours, defined as morning snack, lunch and afternoon snack, which was in line with earlier observations^(^^12^^)^. The total EI of both girls and boys was slightly lower than the reference values for this age group^(^^19^^)^, which is most likely due to a general under-reporting commonly found in this age group^(^^32^^,^^33^^)^.

The children in the present study ate less fruit, vegetables and fish than the FBDG^(^^5^^)^. The same results were found in other studies^(^^14^^,^^17^^,^^18^^)^. The importance of eating fruits and vegetables to promote healthier dietary habits among school children was emphasised with the recent European Union school fruit and vegetable programme in the member states^(^^17^^,^^34^^)^. The Danish children in the present cross-sectional study had a higher intake of fruit during the school hours than the rest of the day, whereas the same difference was not seen for vegetables. The inconsistent results across studies make it difficult to draw conclusions about the impact of the school environment on the total daily intake of fruit and vegetables; besides, some studies have not utilised the hierarchical structure of the data^(^^35^^)^. The children reported eating less meat during the school hours compared with the rest of the day; this meat, however, was eaten as topping on sandwiches at lunch and is often processed meat with a high content of Na and fat. The reported intake of bread was higher during the school hours compared with the rest of the day, which is in accordance with the typical lunch in Danish schools consisting of open sandwiches^(^^6^^)^.

The children in the present study had a considerably lower intake of vitamin D than the level of 10 µg/d as recommended by the NNR2012^(^^19^^)^. Since exposure to sunlight is limited in the Northern European countries especially during winter, oral intake is important. A recent randomised family-based Danish study showed that fortification of milk and bread may be a useful strategy to increase the vitamin D intake also among children in the winter season^(^^36^^)^. Strategies other than fortification could be applied to obtain an increased intake of vitamin D, i.e. campaigns encouraging the importance of eating more fish. An increased fish intake could be part of a strategy to improve vitamin D intake substantially.

No sex differences regarding the energy distribution from total fat, total carbohydrate, added sugar and protein were found, but girls had a lower intake than boys of most foods and nutrients, except for fruit, where girls had a higher intake. These sex differences are also found in other studies^(^^16^^,^^18^^)^ and may be partly explained by different taste preferences for fruit^(^^17^^,^^37^^)^.

For most foods and nutrients there were no differences in intake between the 3rd and 4th graders. However, 3rd graders had a significantly higher intake than 4th graders of fatty fish, dietary fibre, vitamin A, vitamin B_2_, vitamin B_12_, P, Mg, I and K. It cannot be ruled out that the 3rd graders received more parenting aid with the dietary assessment in WebDASC compared with the 4th graders.

Even though direct comparisons with other studies are difficult due to study designs, different assessment methodologies, etc., the overall picture in the present study of low intake of fruit, vegetable, fish and vitamin D, and high intake of fat and sugar are also found in other studies^(^^14^^–^^18^^)^.

The present study is the cross-sectional baseline part of the OPUS School Meal Study. The general strengths and limitations of the OPUS School Meal Study have been described elsewhere^(^^21^^)^. The novel interactive web-based dietary assessment tool developed for children (referred to as WebDASC) was a strength for the participating families, who found it relatively easy to use^(^^22^^)^. It was also a strength that the whole diet was recorded during seven consecutive days and thus making it possible to report on habitual dietary intake. However, it may have been a limitation for a few of the families that they did not have access to a computer with Internet access at home. Besides, the WebDASC was in Danish, which may have been a limitation to the subjects with another language background. It may also have been a limitation that the recording often took place at the end of the day; hence it could possibly be easier to remember meals eaten at the end of the day compared with meals eaten earlier on. It can also be considered as a limitation that the data were collected from August to November instead of all year round. Under- and over-reporting of EI may be a bias that might affect the results of dietary surveys^(^^18^^)^. In the present study, the EI was below the reference values, which could indicate some degree of under-reporting. Our study population was slightly better educated than the rest of the Danish population; however, schools from various socio-economic characteristics were included^(^^21^^)^.

Different strategies to improve children's diet during school hours could be considered in the future. Serving school meals^(^^38^^)^ or improving the quality of the packed lunches targeting both parents and children^(^^39^^)^ could be useful strategies.

Overall, this cross-sectional study showed that compared with the current dietary guidelines and recommendations, 8- to 11-year-old children eat insufficient fruit, vegetable, fish, Fe and vitamin D, but they eat excess amounts meat, saturated fat and added sugar. The study also showed that there is a higher intake of fruit and bread during school hours than outside school hours. This is not the case with, for example, fish and vegetables, and future studies should investigate strategies to increase fish and vegetable intake during school hours.
